# The impact of urbanization and wealth on house dust mite sensitization in children from north-central Nigeria

**DOI:** 10.1186/s13052-022-01348-w

**Published:** 2022-08-19

**Authors:** Chiara Zuiani, Michele Arigliani, Ramatu Zubair, Livingstone Gayus Dogara, Luigi Castriotta, Ashel Dache Sunday, Reward Christopher Audu, Habibah Dadan-Garba, Zakary Sani, Baba Inusa, Paola Cogo

**Affiliations:** 1 Department of Medicine, Unit of Pediatrics, University Hospital of Udine, Piazzale S. Maria Misericordia 1, 33100 Udine, Italy; 2 Department of Pediatrics, Barau Dikko Teaching Hospital, Kaduna State University, Kaduna, Nigeria; 3 Department of Hematology, Barau Dikko Teaching Hospital, Kaduna State University, Kaduna, Nigeria; 4 Institute of Hygiene and Clinical Epidemiology, University Hospital of Udine, Udine, Italy; 5 Paediatric Haematology, Evelina London Children’s Hospital, Guy’s and St Thomas’, National Health Service Foundation Trust, London, UK

**Keywords:** Allergy, Children, House dust mite, Epidemiology

## Abstract

The impact of socio-economic status on the risk of allergy in African children is not clear.

This was a cross sectional study including children aged 6–14 years from urban and rural settings in north-central Nigeria. Participants underwent skin prick tests to house dust mite (HDM) and an interview investigating socio-economic status through the Family Affluence Scale (FAS) based on a score of 0–6.

A total of 346 children were enrolled (52.8% boys; mean age ± SD 9.6 ± 2.0 years), including 142 (41% of total) rural and 204 (59% of total) urban pupils. Prevalence of HDM sensitivity was 2.8% (4/142) in the rural setting and 15.6% (32/204) in the urban setting (*P* < 0.001). Among urban children, frequency of HDM sensitization was 8.6% (7/81) in the lowest socio-economic group (FAS 0–1), 13.1% (8/61) in the intermediate one (FAS 2–3) and 27.4% (17/62) in the highest one (FAS ≥ 4).

Urbanization and increasing wealth are associated with a higher frequency of sensitization to HDM in Nigerian children.

## Introduction

The burden of allergy in sub-Saharan Africa has increased over the last decades [1]. Dust mites are ubiquitous in humid environments and represent one of the most frequent causes of respiratory allergies [2].

The SAFFA study recently showed that living in a rural environment is protective against HDM sensitization in South-African children without atopic dermatitis [3]. Previous evidence from different countries all-over the world showed that a lower socio-economic status (SES) is generally associated with a lower risk of atopy (as opposite to asthma risk) [4] and this seems to be valid also in African children [5]. A protective role of poverty against allergic sensitization would be in keeping with the hygiene hypothesis, according to which better hygiene conditions related to urbanization and higher SES result in reduced exposure to infectious agents in early life, increasing the subsequent risk of allergic sensitization [5].

Previous studies investigating the prevalence of atopy in children from sub-Saharan Africa evaluated only proxies of SES (e.g. type of school attended) [6] but not direct measures of the level of affluence [7], with the risk of selection bias.

This cross-sectional study aimed to compensate the paucity of data regarding the impact of SES on HDM sensitization in children living in sub-Saharan Africa.

We hypothesized that both urbanization and SES would have affected the risk of sensitization to HDM among pupils from north-central Nigeria, possibly reflecting differences in microbial exposure in early life.

## Methods

Data collection was performed in March 2017 in Kaduna State, which is situated in a high plains region in the north-central Nigeria and has a population of around 6 million people (40 in urban and 60% in rural areas). The region has a tropical savannah climate and lush vegetation, mainly characterized by scattered short trees, shrubs and grasses.

Children aged 6–14 years from one private and one public school in Kaduna city and from one rural school approximately 20 km away from town, were enrolled through a convenience sampling. In each school, one class for every level of the primary school (6–12 years) and for the first two levels of the secondary school (13–14 years of age) were involved in the study. The schools were selected according to previous collaborations with the local university hospital. Informed consent from parents and oral assent from the participants was obtained before the assessments. Exclusion criteria were parents or child’s refusal, known primary or secondary immunodeficiency, having taken an anti-histaminic in the 5 days before the assessments, a negative control wheal ≥ 3 mm or a histamine wheal < 3 mm. The study was approved by the Barau Dikko Teaching Hospital Health Research Committee, Kaduna, Nigeria (BDTH-HREC 17–0003-1).

Participants underwent anthropometry measure, skin prick tests (SPT) for HDM and an interview via questionnaire in their first language (Hausa), administered by three local doctors. Skin prick tests were limited to *Dermatophagoides Pteronyssinus* and D. *Farinae* (*ALK-Abello, Horsholm, Denmark*) and were performed on the volar part of the left forearm using 1-mm standardised lancets. Sensitization was defined as a wheal ≥ 3 mm for the tested allergen and histamine after 15 min, whilst negative control had a wheal < 3 mm. The questionnaire investigated SES, through a slightly modified version of the intentionally validated Family Affluence Scale (FAS) [7], based on collated score for number of computers (1 point for each computer up to 3), motorcycle (1 point) or car (2 points) ownership, and whether the child had his own bedroom (1 point). Socio-economic status was classified as high (FAS ≥ 4), intermediate (FAS 2–3) and low (FAS 0–1). Clinical history was not investigated, since we could not interview parents.

Based on the results of an interim analysis on the first 100 participants, a final sample size of at least 139 urban children and 139 rural counterparts would have provided 80% power at the 5% significance level to detect a prevalence of HDM sensititation of 10 in the formers and 2% in the latters.

Group differences were tested through unpaired *t* test and *chi-squared* test as appropriate, using the high SES urban group (FAS ≥ 4) as standard for comparison. A *p*-value < 0.05 was considered as statistically significant. Analyses were conducted using STATA software (StataCorp. 2014. Stata Statistical Software: Release 14. College Station, TX: StataCorp LP).

## Results

A total of 463 children were invited to take part to the study. Of these, 110 were not included as families or themselves refused (23.7% of total), whereas 7 were excluded because of negative control wheal ≥ 3 mm or a histamine wheal < 3 mm at SPT. Distribution by sex and age of these children was comparable (data not shown) with that of the 346 children included in the final analysis (52.8% boys; mean age ± SD 9.6 ± 2.0 years). The final study population included 142 rural children (41%) and 204 urban children (59%) (Table 1). All the pupils with high SES (FAS ≥ 4; 62/346, 18%) attended the private urban school. Rural children and urban children with low SES (FAS 0–1) had markedly lower anthropometry z-scores than those in the high SES group (FAS ≥ 4) (Table 1).

**Table 1:** Anthropometry, socio-economic status (SES) and prevalence of house dust mite sensitization in 346 pupils aged 6–14 years from Kaduna state, Nigeria

	RURAL^a^			URBAN			
	Low SES (FAS 0–1)	Medium SES (FAS 2–3)	Tot	Low SES (FAS 0–1)	Medium SES (FAS 2–3)	High SES (FAS ≥ 4)	Tot
Subjects, n (% of tot)	113 (80)	29 (20)	142	81 (40)	61 (30)	62 (30)	204
Age, yr ± SD	9.3 ± 1.9	9.7 ± 2.4	9.4 ± 2.0	10.3 ± 2.2	9.4 ± 1.7	9.4 ± 1.7	9.8 ± 2.0
Height z-score ± SD	-1.56 ± 0.97	-1.23 ± 1.16	-1.50 ± 1.0	-1.24 ± 1.42	0.19 ± 1.53	1.03 ± 1.02	-0.12 ± 1.66
BMI z-score ± SD	-0.93 ± 0.90	-0.74 ± 0.85	-0.90 ± 0.9	-1.20 ± 1.40	-0.37 ± 1.43	0.70 ± 1.34	-0.37 ± 1.60
House dust mite SPT positivity^b^ n (% of pupils in the SES group)	4 (3.5%)	0	4 (2.8%)	7 (8.6%)	8 (13.1%)	17 (27.4%)^c^	32 (15.6%)

*Abbreviations*: *SES* Socio-economic status, *FAS* Family Affluence Score, *BMI* Body mass index, *SD* Standard deviation, *SPT* Skin prick tests

^a^No children with high SES (FAS ≥ 4) in the rural setting

Height z-score and BMI z-score values based on WHO 2007 growth reference (https://www.who.int/growthref/en/)

^b^wheal ≥ 3 mm for the tested allergen and histamine plus negative control with a wheal < 3 mm 15 min after the SPT

^c^*P* (for differences in frequencies of HDM sensitization): 0.04 compared to urban medium SES group (FAS 2–3) and = 0.003 compared with urban low SES group (FAS 0–1)

Overall prevalence of HDM sensitization was 10.4% (36/346), with 8.6% (30/346) positive to *D. Pteronyssinus* and 7.2% (25/346) to *D. Farinae*. The frequency of SPT positivity to HDM was 15.6% (32/204) in urban children (including 30 children sensitized for *D. Pteronyssinus* and 32 sensitized for *D. Farinae*) and 2.8% (4/142) in rural children (including 4 children sensitized for both *D. Pteronyssinus* and *D. Farinae*) (*P* < 0.001). Among pupils living in the urban setting, there was a descending gradient in HDM sensitization according to decreasing SES, ranging from 27.4% (17/62) in the highest SES group (FAS ≥ 4), to 13.1% (8/61) in children with intermediate SES (FAS 2–3, *P* = 0.04 compare with high SES group), to 8.6% (7/81) in the poorest children (FAS 0–1, *P* = 0.003 compare with high SES group) (Table 1).

## Discussion

We found a very low frequency of HDM sensitization in children living in a rural setting (2.8%) and a positive association between the level of affluence and HDM sensitization in children living in an urban area in Nigeria.

The prevalence of SPT positivity to HDM in urban pupils from north-central Nigeria (15.6%) was slightly lower than previously reported in children of similar age range attending public and private schools in the capital city, Lagos, in the south of the country (20.7%) [8]; however the difference resulted not statistically significant (*P* = 0.05) when the populations of the two studies were compared using a chi-squared test.

*Addo-Yobo *et al. previously reported a descending gradient in the prevalence of SPT positivity to HDM in Ghanaian children (age range 9–16 years) attending a “urban rich”, a “urban poor” and a rural school [6]. Provided that the type of school attended is only a proxy of SES and could misclassify some of the participants, these findings are consistent with ours.

We can only speculate on the mechanisms that determined our findings. In South-Africa, farm animal exposure was the strongest protective factor against allergy in children living in a rural environment [9]. Regular exposure to livestock was also present in rural children included in this study and might have contributed to the low prevalence of HDM sensitivity found in this group.

Moreover, it is likely that rural pupils were exposed to a more diverse environmental microbiome in early life and, possibly, to a higher rate of chronic helminths infection, compared with their counterparts living in the urban environment. These phenomena might have strengthen regulatory mechanisms of the immune system that limit Th-2 mediated hypersensitivity responses to aeroallergens. In urban children increasing SES were probably associated with better hygiene condition, with exposure to a narrower variety of bacteria, endotoxins and parasites in early life, resulting in a higher risk of allergic sensitization.

Among limitations: participants were selected by convenience sampling and we cannot exclude that this biased the results in some ways. Moreover, we could not obtain clinical information from the participants, including personal or family history of allergy, and we could not measure exposure to HDM or to other environmental factors that might have influenced results, such as microbial exposure in early life. A strength is that we assessed HDM sensitization in relation to SES, using a validated measure [7].

In conclusion, this study showed that urbanization and increasing level of affluence are associated with a higher frequency of sensitization to HDM in children living in north-central Nigeria. Future studies should further investigate the mechanisms determing such differences, with special attention at early life determinants.

## Data Availability

The datasets used and/or analysed during the current study are available from the corresponding author on reasonable request.

## Abbreviations

FAS: Family affluence scale
HDM: House dust mite
SES: Socio-economic status
SPT: Skin prick tests
